# Genetic Analysis of Avian Influenza Viruses: Cocirculation of Avian Influenza Viruses with Allele A and B Nonstructural Gene in Northern Pintail (*Anas acuta*) Ducks Wintering in Japan

**DOI:** 10.1155/2012/847505

**Published:** 2012-12-25

**Authors:** Alam Jahangir, Sakchai Ruenphet, Nadia Sultana, Dany Shoham, Kazuaki Takehara

**Affiliations:** ^1^Laboratory of Zoonoses, School of Veterinary Medicine, Kitasato University, Towada 034-8628, Japan; ^2^Animal Health Research Division, Bangladesh Livestock Research Institute, Savar, Dhaka 1341, Bangladesh; ^3^Tokyo University of Agriculture and Technology, Tokyo 183-0057, Japan; ^4^Begin-Sadat Center for Strategic Studies, Bar-Ilan University 52900, Ramat Gan, Israel

## Abstract

The pandemic influenza virus strains of 1918 (H1N1), 1957 (H2N2), 1968 (H3N2), and 2009 (H1N1) have genes related to avian influenza viruses (AIVs). The nonstructural (NS) gene of AIVs plays a significant role in host-viral interaction. However, little is known about the degree of diversity of this gene in Northern pintail (*Anas acuta*) ducks wintering in Japan. This study describes characteristics of pintail-originated H1N1, H1N2, H1N3, H5N2, H5N3, H5N9, and H7N7 viruses. Most of the viruses were revealed to be avian strains and not related to pandemic and seasonal flu strains. Nevertheless, the NP genes of 62.5% (5/8) viruses were found closely related to a A/swine/Korea/C12/08, indicating exchange of genetic material and ongoing mammalian-linked evolution of AIVs. Besides, all the viruses, except Aomori/422/07 H1N1, contain PSIQSR∗GLF motif usually found in avian, porcine, and human H1 strains. The Aomori/422/07 H1N1 has a PSVQSR∗GLF motif identical to a North American strain. This findings linked to an important intercontinental, Asian-American biogeographical interface. Phylogenetically all the viruses were clustered in Eurasian lineage. Cocirculation of allele A and B (NS gene) viruses was evident in the study implying the existence of a wide reservoir of influenza A viruses in pintail wintering in Japan.

## 1. Introduction

Influenza A virus infections in birds account for important inputs into the evolutionary porcine-human complex of this prominent anthropozoonotic pathogen. Among influenza A viruses, which broadly exhibit 17 HA and 9 NA antigenic subtypes, only three haemagglutinin (HA) subtypes (H1, H2, and H3) and two neuraminidase (NA) subtypes (N1 and N2) have circulated widely in swine and human populations since the 20th century [1]. Viruses from waterfowl reassorted with existing human and/or porcine influenza viruses to generate the 1957, 1968 [2], and 2009 (Novel swine-origin influenza A (H1N1) virus investigation team, 2009) pandemic influenza viruses and may expectably play a similar role in the creation of future pandemic viruses. In addition, on multiple occasions, it has been evident that avian influenza viruses (AIVs), chiefly the subtypes H5N1, H7N7, and H9N2, directly transmitted from birds to humans [3, 4]. Avian-originated H1N1, H3N2, H5N1, and H9N2 viruses have been recovered from pigs in Asia, Europe, and Canada [5–7]. Furthermore, H2N3 avian virus reassortants were isolated from pigs in the United States [8].

Pigs have been postulated, hence, to be the ultimate “mixing vessels” for mammalian influenza viruses and AIVs and can play an important role in the genetic reassortment of influenza viruses. Therefore, detection and characterization of AIVs, especially those subtypes having the potential to transmit to mammals, including pigs and humans, are significant. Avian H7N7 and H3N8 strains are contracted and circulated by horses, as well, and an equine H3N8 virus apparently was involved in the formation of the pandemic H3N2 virus, and perhaps the H3N8 pandemic virus of 1889 [9]. 

The genome of influenza A viruses consists of eight different segments of single-stranded negative-sense RNA. Most gene segments encode for one protein; whereas the gene segments 2 (polymerase basic1—PB1), 7 (matrix—M), and 8 (nonstructural—NS gene), each encodes one additional protein from alternatively spliced mRNA. Among the proteins, 9 are structural, while the rest two, namely PB1-F2 and NS1, are nonstructural. Thirteen genes have already been identified all over. Aquatic birds are the primary reservoir of type A influenza viruses and are classified into various antigenic subtypes, based on their two surface glycoproteins, the HA, and the NA. So far, seventeen HA (H1–H17) and nine NA subtypes (N1–N9) have been identified from avian species [10], representing the entire pool of influenza A viruses known today. Most of them are not found or uncommon within mammalian hosts. Although largely shaped by the HA, the virulence of influenza A viruses is polygenic in nature. H5 and H7 influenza viruses with multiple basic amino acids nearby to the cleavage site of the HA glycoprotein exhibit a wide range of tissue tropism and lead to systemic disease in chickens with fatality [11].

It has been demonstrated that the amino acids at positions 627 and 701 of the polymerase basic 2 (PB2) protein influence the outcome of infection in mice [12, 13]. Several studies have reported that the NS1 protein is also associated with the virulence of influenza viruses [14, 15]. The glutamic acid at position 92 of NS1 of H5N1 influenza virus confers virulence and resistance to antiviral cytokines in pigs [16]. In spite of those well-known, meaningful functions of the NS gene concerning virulence and escape from host cytokine response, the degree of variation in the NS gene pool of AIVs in their natural reservoirs, particularly in the Northern pintail (*Anas acuta*) (thereafter referred to as pintail (s)) ducks, a globally major influenza host wintering in Japan, is poorly studied. In this paper, we focused on genetic analysis of H1N1, H1N2, and H1N3 strains of AIVs that we isolated from apparently healthy migratory pintails wintering in Japan [17, 18]. Also, we analyzed the NS genes of H5 and H7 subtypes, in addition to the above-mentioned AIVs. Moreover, polymerase acid (PA), nucleoprotein (NP), M, and NS genes of H5N9 strain, an uncommon antigenic combination which we isolate for the first time in Asia [19], were further looked into in the present study.

## 2. Material and Methods

### 2.1. Viruses

Viruses used in this study (Table 1) were of low pathogenicity and isolated in embryonated chicken eggs (ECE) from fecal materials of migratory, apparently healthy pintails wintering in Japan [17, 18]. Working stocks of viruses were prepared by 3rd passage in ECE, and allantoic fluid was harvested at 3 days after inoculation (dpi) and stored at −80°C.

### 2.2. RNA Extraction and RT-PCR

Total viral RNA was extracted from infected allantoic fluid using the Isogen-LS (Nippongene, Tokyo, Japan), in accordance with the manufacturer's instructions. Reverse transcription was carried out with the Uni12 primer (5′-AGC AAA AGC AAG G-3′) and MMLV reverse transcriptase (GeneAmp RNA PCR Kit, Applied Biosystems, Tokyo, Japan), followed by full length amplification of each gene segment, as described earlier [20]. The PCR product was purified using QIAquick Gel Extraction Kit (Qiagen, Valencia, CA), according to the manufacturer's instructions.

### 2.3. Gene Sequencing

The PCR product was sequenced by dideoxy chain terminating method, using Dye Terminator Cycle Sequencing FS Ready Reaction Kit (Perkin-Elmer Applied Biosystems, Japan). Sequencing was carried out with the same primers used to amplify the gene and continued with subsequently designed sequence-specific primers. Nucleotide sequences were determined using an automated DNA sequencer (ABI 310 DNA sequencer, Applied Biosystems, Foster City, CA), edited and assembled with GENETYX-Mac (version 10.0; Software Development Corp., Tokyo, Japan).

### 2.4. Sequence and Phylogenetic Analysis

Multiple sequence alignments and processing were performed with the Molecular Evolutionary Genetics Analysis (MEGA) version 4.1.0 software, with an engine based on the ClustalW algorithm [21]. Blast searches were used to retrieve the homologous sequences from the GenBank database. The phylogenetic analyses were performed using neighbor joining tree inference analysis, with the 1000 bootstrap replications, to assign confidence levels to branches. Nucleotide sequences of entire open reading frame (ORF) of each gene segment (except PB1 gene of A/northern pintail/Aomori/1130/08 H1N3 (Aomori/1130/08 H1N3)) were used in the phylogenetic analysis. The PB1 gene of Aomori/1130/08 H1N3 was partially 1350 base pair (1-1350 bp) sequenced and used in the phylogenetic analysis. Furthermore, the HA gene of A/northern pintail/Akita/1364/08 H1N2 (Akita/1364/08 H1N2) was sequenced partially 765 bp (760-1524) and was not included in the phylogenetic analysis.

### 2.5. GenBank Accession Number

The nucleotide sequences generated in this study have been deposited in GenBank and are available under accession number AB546149–AB546193.

## 3. Results and Discussion

As a part of longitudinal virological studies, we have isolated various subtypes of AIVs from fecal materials of migratory, apparently healthy pintails wintering in Tohoku district, Japan [17, 18]. Of these, H1N1, H1N2, and H1N3 viruses were characterized genetically. For phylogenetic relationships of NS gene, the latter from viruses bearing H1, H5, and H7 HA was sequenced and compared with viruses from GenBank.

### 3.1. Genetic and Phylogenetic Analysis of HA Genes

**Figure 1:**
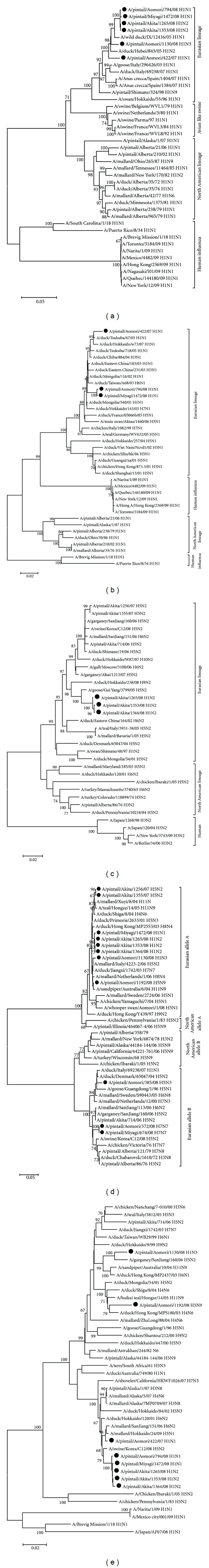
Phylogenetic analysis of avian influenza viruses isolated from Northern pintails in Tohoku district, Japan. Included are different segment of AIVs. Individual tree was generated using neighbor-joining method and 1,000 replications of bootstrap resampling. The number at each branch point indicates percentage probability that the resultant topology is correct. The ORF of each gene segment (a) H1 (1701 bp), (b) N1 (1410 bp), (c) N2 (1410 bp), (d) NS (838 bp), and (e) NP (1497 bp) was employed to generate phylogram. Viruses of the present study were marked by black circle (●) in the tree. Strains with no host were assumed to human isolates.

The sequenced HA genes (*n* = 6) of H1 subtype with different NA subtype combinations (N1, N2, and N3) isolated from pintails during 2007-2008 were closely related to each other (Figure 1(a); Table 2). The nucleotide and amino acid sequence identities were found to range from 96.5 to 100% and 98.0 to 100%, respectively. Phylogenetically, the viruses were clustered with the viruses from Eurasian countries, mostly with viruses from China, namely A/duck/Hebei/843/05 H1N2 and A/wild duck/JX/12416/05 H1N1 (>97% nucleotide homology) (Figure 1(a)). The HA genes of sequenced viruses were grouped into the h1.1.2 (subtype H1) [22]. Viruses of the present study had a sister group relationship with the Japanese H1 viruses including A/pintail/Shimane/324/98 H1N9 isolated earlier. North American strains, and swine, human seasonal and pandemic H1 strains integrated sharply in different branches. It means that although bearing the same antigenic subtype (H1N1) and obtained during 2007-2008, our isolates were unrelated to the porcine-derived 2009 pandemic and seasonal flu strains.

### 3.2. Genetic and Phylogenetic Analysis of NA Genes

Like HA genes, the nucleotide homologies of the NA genes of the isolates with each other were 96.8–99.8% and 99.7–99.9% for N1 (*n* = 3) and N2 (*n* = 3), respectively, for each subtype. On the other hand, the amino acid sequence identities were found to range from 97.4 to 99.8% (N1) and 99.4–99.8% (N2). A duck-originated Japanese strain obtained 2 years earlier, A/duck/Tsukuba/67/05 H1N1 (Tsukuba/67/05 H1N1) was found most closely related to one of the isolates—A/northern pintail/Aomori/422/07 H1N1 (Aomori/422/07 H1N1) (>98% homology at both nucleotide and amino acid sequences)—of the present study, while two other viruses have maximum homology to a Mongolian strain, A/duck/Mongolia/116/02 H1N1 (Mongolia/116/02 H1N1), isolated in 2002 (>98% homology at both nucleotide and amino acid sequences). For N2 NAs, a Chinese strain, namely A/goose/Gui Yang/3799/05 H5N2 (Gui Yang/3799/05 H5N2) isolated from goose in 2005 was found rather closely related (>98% nucleotide and amino acid identity). The N3 NA gene of Aomori/1130/08 H1N3 has >99% nucleotide and amino acid homology to a duck strain, namely A/duck/Niigata/514/06 H5N3 (Niigata/514/06 H5N3) isolated in 2006 in Japan (Table 3). Principally, the NA genes of the sequenced viruses were clustered with viruses of Eurasian origin (Figures 1(b) and 1(c)). More specifically they can be grouped into sublineages n1.1.8 (N1 subtype) and n2.1.6 (subtype N2) [22]. NA gene of Aomori/422/07 H1N1 strain branched separately from other strains isolated by us in 2008 (Figure 1(b)), the N2 NA genes of which were all integrated into a single branch represented by a H5N2 strain, Gui Yang/3799/05, which was isolated in China in 2005 (Figure 1(c)). Like H1 HA genes, the N1 NA genes were distinctly separated from pandemic strains (Figure 1(b)).

### 3.3. Genetic and Phylogenetic Analysis of NS Genes

A remarkably diverse variation in the nucleotide sequence identities (50–100%) was found among the NS genes of the sequenced viruses (Table 4). When analyzed phylogenetically, they were distinctly branched into two branches, allele A and B [23], and clustered with Eurasian origin viruses (Figure 1(d) and Table 4). Nucleotide sequence identities of NS genes within allele A and within allele B were 97.7–100% and 94.4–100%, respectively. However, the divergence between the two alleles was approximately 50%, regardless of their isolation time and geographical location. The majority of the viruses of the present study were branched with allele A viruses represented with/by A/mallard/Sweden/2724/06 H5N1 and A/duck/Hong Kong/Y439/97 H9N2 (Hong Kong/Y439/97 H9N2). In contrast, A/northern pintail/Aomori/372/08 H7N7 (Aomori/372/08 H7N7), Aomori/385/08 H5N3, and A/northern pintail/Miyagi/674/08 H7N7 (Miyagi/674/08 H7N7) viruses were clustered with allele B viruses represented with/by A/goose/Guangdong/1/96 H5N1 (Guangdong/1/96 H5N1). As for allele A, two pairs—Akita/1256/07 H5N2 and Akita/1355/07 H5N2, alongside with Akita/1353/08 H1N2 and Akita/1364/08 H1N2 viruses—were found 100% homologous, while in allele B Aomori/372/08 H7N7 and Miyagi/674/08 H7N7 viruses were found to be entirely identical.

Significantly, the NS1 genes of influenza viruses in general have been divided into two alleles: A and B [23]. Regardless of some exceptions, all influenza viruses circulating in mammalian species and many viruses from avian species comprise the allele A, while allele B is found within only AIVs. The NS1 gene of the majority of highly pathogenic avian influenza (HPAI) H5N1 viruses isolated from humans since 1997 is affiliated with allele A. In contrast, the NS1 gene of the Guangdong/1/96 virus, which was the source of the initial HPAI H5N1/1997 HA gene, belonged to allele B [24]. The NS1 genes of viruses of the present study—all LPAI—belong either to allele A or allele B. Curiously, it was pointed out that some strains of the HPAI H5N1 virus were preserved genetically unchanged from 1997 up to 2005 [25]. In that connection, perhaps, it is of note that the NS gene of some viruses, for example Aomori/372/08 H7N7 and Miyagi/674/08 H7N7 of this study have only <3% nucleotide disparity from that of a Russian strain, namely A/duck/Chabarovsk/1610/72 H3N8 (Chabarovsk/1610/72 H3N8), isolated in 1972. The NS gene of one of the viruses we had isolated from pintail ducks, namely Akita/714/06 H5N2, has 98.3% nucleotide homology to the same Russian isolate Chabarovsk/1610/72 H3N8 [26]. It means that the ongoing mutation rate during about 35 years was only about 1.7%, which is markedly less than the expected one. This may imply that throughout about a third of that 35 years period the NS gene has been somehow conserved, considering that the estimated NS gene evolutionary rates for avian, classic swine, and equine lineages are 0.84–1.27 nucleotide changes per site per year [27], and even higher, within both wild and domestic avian host species [28]. Basically, the two reasons thought to possibly account for unexplained genetic conservation in influenza A viruses are cross contamination, or interference with vaccine strains [29]. The strain Chabarovsk/1610/72 or related strains are not held in our laboratory, and interference with a vaccine strain seems unlikely, in that case. Abiotic environmental virus preservation has been suggested as an alternative explanation [30]. These, together with the location of Chabarovsk being close to the regular migration route of pintails between Japan and Siberia, have to further be looked into. 

Prevalence of allele A and B viruses in their natural hosts has been reported to vary spatially, as well. Generally, allele B viruses were reported to be less common in their natural hosts than allele A. Out of 11 NS genes sequenced in this study, 72.7% (8/11) were classified as allele A, and 27.3% (3/11) as allele B. In Asia, the prevalence of allele B viruses in all avian species has been reported to be only 15% [31], which is markedly lower than found in this study. However, the prevalence rate of allele B viruses in North American free flying birds has been documented to be 30%, similar to that observed in pintails in our study [32], but higher than found in Northern European mallards (13%; 6 out of 45) [31]. This might be due to the migration pattern of pintails sampled in our study. Most pintails which winter in Japan originate from eastern Russia [33, 34]. Besides, pintails marked in North America have been recovered during winter in Japan and vice versa [35, 36]. Moreover, some pintails migrate from North America to eastern Russia [37], where they could come into contact with birds that migrate from six continents, including Asian wintering sites [38]. Furthermore, from the recent satellite telemetry data, provided by the United States Geological Survey (USGS) and the US Fish and Wildlife Service, it is evident that pintails marked during winter in Japan move to Alaska through Russia and return to Japan, following the same route (Alaska Science Center, Movements of Northern Pintail ducks and Whooper Swans marked with satellite transmitters in Japan http://alaska.usgs.gov/science/biology/avian_influenza/pintail_movements_virus.php. Thus, inter- and intra continental exchange of genes and genomes of AIV could occur through congregation of North American and Asian migrants at shared summer habitats in eastern Russia, or by means of pintails that migrate between North American nesting grounds and Japanese wintering grounds (assessment of virus movement across continents: using Northern Pintails (Anas acuta) as a test. http://alaska.usgs.gov/science/biology/avian_influenza/migration_ecology.php. Also the number of samples may possibly have effect on the results found in this study.

### 3.4. Genetic and Phylogenetic Analysis of NP Genes

The NP genes of the sequenced viruses have 90.4–99.9% nucleotide homologies among each other, regardless of their temporal and spatial differences in isolation (Table 5). Broadly, viruses isolated from Eastern Hemisphere have high homologies to the NP genes of the viruses of this study (97.2 to 99.5% nucleotide identity). It is noteworthy that the NP gene of the majority of viruses (75% of 8 viruses) investigated in this study had a high homology to a porcine strain, namely A/swine/Korea/C12/08 H5N2 (Korea/C12/08 H5N2) isolated in 2008, suggesting that the NP genes of these viruses and the Korea isolate might have a common ancestral origin. Similar to NP gene of H1 viruses, the HA genes of some H5N2 viruses isolated by us during 2006-2007 were found to be closely related to this Korean virus [19, 26]. In conjunction, biogeographically and phylogenetically, other related strains, namely A/garganey/San Jiang/160/06 H5N2, A/baikal teal/Hongze/14/05 H11N9, and A/duck/Hong Kong/MPS180/03 H4N6 originated from China, and one strain, A/mallard/Hokkaido/24/09 H5N1, from Japan. For NP gene viruses from both Eurasian countries and North America were clustered with the viruses of the present study (Figure 1(e)). The nucleotide sequence disparity between viruses of this study and North American viruses clustered together ranged from 1.4 to 9.3%. Especially, and not by chance, in all likelihood, Aomori/422/07 H1N1 has 98.6% nucleotide identity to the strain A/pintail/Alaska/1/07 H3N8, meaning isolated from the same host in the same year. In that case, viruses of North American lineage integrated distinctly from those of Eurasian lineage, suggesting, in light of the mentioned high NP gene homology, intercontinental gene exchange through reassortment, rather than whole genome intercontinental conveyance. Such interfaces are at any rate significant, evolutionarily and epidemiologically.

### 3.5. Molecular Features of HA and NA

The HA amino acid sequences deduced from nucleotide sequences analysis revealed that all the viruses contain seven potential N-linked glycosylation sites (5 in HA1 and 2 in HA2) throughout the HA molecule. Except for Aomori/422/07 H1N1, the amino acid motif of the HA0 cleavage site of all the sequenced H1 viruses was PSIQSR*GLF (*cleavage point), which is common in all avian, porcine, and human viruses. Aomori/422/07 H1N1 contains a rare amino acid motif, PSVQSR*GLF. Among the H1 avian strains isolated globally and sequences reported in GenBank during 1980–2009, only one strain, namely A/mallard/New York/170/82 H1N2 has identical (PSVQSR*GLF) amino acids. However, Aomori/422/07 H1N1 solely clustered with the Eurasian viruses (Figure 1(a)), implying, ostensibly that it has no relation with the mallard-originated New York strain and evolves individually. Still, the extreme rareness of the mentioned motif might possibly be supportive of genetic recombination event that perhaps took place within collocated ducks from North America and Asia. The HA molecules of all of the sequenced H1 viruses contain residues Gln (Q) and Gly (G) at positions 240 and 242 (H1 numbering), respectively, which indicate the avian receptor specificity.

On the other hand, the amino acid sequence analysis of NAs revealed that all the viruses contain full length NA protein with seven potential N-linked glycosylation sites in N1 and N2 known to be conserved in wild ducks [39]. In all NA molecules, a residue His (H) was found at position 274 (N2 numbering), thereby indicating sensitivity to Oseltamivir or Zamamivir [40]. Besides, typical catalytic sites, framework sites as well as other specific regions were found completely conserved as described previously [41]. Residue position 198 in N3 NA contains Asn (N) instead of Asp (D) and is also shown to be conserved [41]. The N1 and N2 NA of the sequenced viruses possessed E119 and R292 amino acid residues, indicating that the commonly mutated residues in NA inhibitor-resistant viruses were not present [42].

### 3.6. Molecular Features of NS1

Deletion of several amino acids in the NS1 gene has been observed more frequently in AIVs in recent years, a feature of possible adaptation of these viruses to poultry [24, 43]. The viruses sequenced in our study contained full length NS genes, indicating basically wild bird strains. Nevertheless, all isolates possessed residue Ala (A) position 149, which is important for replication of viruses in chickens [24]. Also, residue Asp (D) instead of Glu (E) was found at position 92 of the NS1, which is reported to be involved in modulation of cytokine response, and has been associated with the high virulence exhibited by HPAI H5N1 viruses in pigs [16]. All the sequenced viruses contain a PDZ (postsynaptic density, PSD-95; discs large, Dlg; zonula occludens-1, ZO-1) domain ligand at the C terminus of NS1 (ESEV-COOH), which plays an important role in many key signaling pathways of viral replication [44].

### 3.7. Relation between Avian H1N1 Strains and Pandemic and Seasonal Influenza Strains

When compared to pandemic and seasonal influenza viruses, the HA genes' nucleotide identity of viruses of the present study was found to range from 67.1 to 76.4%, while amino acid sequence homology was found 81.8–92.1% (Table 6). In difference, for NA gene, a somewhat higher nucleotide and amino acid identity was found (79.3–86.5% nucleotide and 84.9–92.5% amino acid sequence identities). Not only the HA and NA genes but also other internal genes of pandemic and seasonal flu strains were clustered distinctly from those of our isolates, suggesting no relation between these viruses. Basically, genomic analyses of the last four pandemic strains showed that the genes contributed by avian strains are those that encode for the polymerases and surface antigens, as follows:1918—PB1 and PA; 1957—PB1, HA, and NA; 1968—PB1 and HA; 2009—PB2 and PA. This means that among the presently prevailing pandemic and seasonal strains, only the seasonal H3N2 has past affinity to avian HA, when it originally formed as a then pandemic strain in 1968, thereafter considerably drifting genetically for already 43 years. Independent drift probably took place during that period of time within the precursor avian H3 HA gene in bird populations.

## 4. Conclusion

In conclusion, our genetic analysis suggests that the sequenced viruses were to an appreciable degree characteristic of the pintail populations we sampled, which typically winter in Japan. Except in one strain, the HA0 cleavage site of H1 was found as usually found in all avian, porcine, and human viruses. The residues that compose the catalytic and framework sites of the NA enzyme were completely conserved in the studied viruses. The NP gene of the majority of the strains sequenced in this study (5/8) was related to that of the porcine strain Korea/C12/08 H5N2. Thus, it may be presumed that our isolates evolved through reassortment process during the cocirculation of these strains. Our findings clearly demonstrate that two distinct gene pools, corresponding to both NS allele A and B, were present within the pintail populations wintering in Japan. It is noteworthy that some strains contain NS gene highly related to a duck isolate obtained in Russian in 1972, about 35 years prior to our isolate, indicating significant gene conservation. It is also concluded that although bearing the same antigenic subtype (H1N1) and obtained during 2007-2008, our isolates were unrelated to 2009 pandemic and seasonal flu strains. 

## Figures and Tables

**Table 1:** Avian influenza viruses used in the present study^a^.

Virus	Gene segment sequenced	HA0 cleavage site (*cleavage point)^b^
A/pintail/Aomori/422/07 H1N1	HA, NP, NA, and M	PSVQSR*GLF
A/pintail/Aomori/794/08 H1N1	HA, NP, NA, and M	PSIQSR*GLF
A/pintail/Miyagi/1472/08 H1N1	HA, NP, NA, M, and NS	PSIQSR*GLF
A/pintail/Akita/1265/08 H1N2	HA, NP, NA, M, and NS	PSIQSR*GLF
A/pintail/Akita/1353/08 H1N2	HA, NP, NA, M, and NS	PSIQSR*GLF
A/pintail/Akita/1364/08 H1N2	HA^c^, NP, NA, M, and NS	PSIQSR*GLF
A/pintail/Aomori/1130/08 H1N3	PB2, PB1^c^, PA, HA, NP, NA, M, and NS	PSIQSR*GLF
A/pintail/Aomori/1192/08 H5N9	PA, NP, M, and NS	—^d^
A/pintail/Akita/1256/07 H5N2	NS	—
A/pintail/Akita/1355/07 H5N2	NS	—
A/pintail/Aomori/385/08 H5N3	NS	—
A/pintail/Aomori/372/08 H7N7	NS	—
A/pintail/Miyagi/674/08 H7N7	NS	—

^a^The viruses were isolated from apparently healthy migratory pintails wintering in Japan. Viruses were isolated in embryonated chicken eggs. The entire open reading frame (until otherwise mentioned) of each gene segment was sequenced from PCR product; ^b^the amino acid sequences were deduced from nucleotide sequences; ^c^partial sequences—for H1 HA 765 base pair (760-1524) and for PB1 1350 base pair (1-1350); ^d^not applicable.

**Table 2:** Percentage identity (%) of nucleotide sequences of the HA genes of avian influenza viruses: intrahomology and interhomology with interrelated sequences from GenBank.

Virus	Identity^a^ (%)	Highest identity^b^ (%)
Subtype H1		
A/pintail/Aomori/422/07 H1N1	96.5–100	A/duck/Hebei/843/05 H1N2 (97.2)
A/pintail/Aomori/794/08 H1N1		A/wild duck/JX/12416/05 H1N1 (>97.3)
A/pintail/Miyagi/1472/08 H1N1		
A/pintail/Akita/1265/08 H1N2		
A/pintail/Akita/1353/08 H1N2		
A/pintail/Aomori/1130/08 H1N3		

^a^Nucleotide sequence identity among the isolates; ^b^identity with genes of viruses from GenBank.

**Table 3:** Percentage identity (%) of nucleotide sequences of the NA genes of avian influenza viruses: intrahomology and interhomology with interrelated sequences from GenBank.

Virus	Identity^a^ (%)	Highest identity^b^ (%)
Subtype N1		
A/pintail/Aomori/422/07 H1N1	96.8–99.8	A/duck/Tsukuba/67/05 H1N1 (98.8)
A/pintail/Aomori/794/08 H1N1		A/duck/Mongolia/116/02 H1N1 (>98.6)
A/pintail/Miyagi/1472/08 H1N1		

Subtype N2		
A/pintail/Akita/1265/08 H1N2	99.7–99.9	A/goose/Gui Yang/3799/05 H5N2 (>98.2)
A/pintail/Akita/1353/08 H1N2		
A/pintail/Akita/1364/08 H1N2		

Subtype N3		
A/pintail/Aomori/1130/08 H1N3		A/duck/Niigata/514/06 H5N3 (99.6)

^a^Nucleotide sequence identity within each gene segment of our isolates; ^b^identity with genes of viruses from GenBank.

**Table 4:** Percentage identity (%) of nucleotide sequences of the NS genes of avian influenza viruses: intrahomology and interhomology with interrelated sequences from GenBank.

Virus	Identity^a^ (%)	Highest identity^b^ (%)
Segment 8 (NS)		
A/pintail/Akita/1256/07 H5N2		A/mallard/Xuyi/8/04 H11N? (99.4)
A/pintail/Akita/1355/07 H5N2		
A/pintail/Aomori/372/08 H7N7		A/duck/Chabarovsk/1610/72 H3N8 (97.4)
A/pintail/Miyagi/674/08 H7N7		
A/pintail/Aomori/385/08 H5N3		A/duck/Denmark/65047/04 H5N2 (98.3)
A/pintail/Aomori/1130/08 H1N3	50–100	A/mallard/Italy/4223-2/06H5N2 (>99.3)
A/pintail/Aomori/1192/08 H5N9		
A/pintail/Akita/1265/08 H1N2		A/mallard/Italy/4223-2/06H5N2
		A/duck/Primorie/2633/01 H5N3 (both viruses 98.9)
A/pintail/Akita/1353/08 H1N2		A/duck/Primorie/2633/01 H5N3 (>98.9)
A/pintail/Akita/1364/08 H1N2		
A/pintail/Miyagi/1472/08 H1N1		

^a^Nucleotide sequence identity within each gene segment of our isolates; ^b^identity with genes of viruses from GenBank.

**Table 5:** Percentage identity (%) of nucleotide sequences of nucleoprotein (NP) coding genes of avian influenza viruses: intrahomology and interhomology with interrelated sequences from GenBank.

Virus	Identity^a^ (%)	Highest identity^b^ (%)
Segment 5 (NP)		
A/pintail/Aomori/422/07 H1N1	90.4–99.9	A/garganey/SanJiang/160/06 H5N2
		A/mallard/Hokkaido/24/09 H5N1 (both viruses 99.5)
A/pintail/Aomori/794/08 H1N1		A/swine/Korea/C12/08 H5N2 (>98.8)
A/pintail/Akita/1265/08 H1N2		
A/pintail/Akita/1353/08 H1N2		
A/pintail/Akita/1364/08 H1N2		
A/pintail/Miyagi/1472/08 H1N1		
A/pintail/Aomori/1130/08 H1N3		A/garganey/SanJiang/160/06 H5N2 (97.7)
A/pintail/Aomori/1192/08 H5N9		A/baikal teal/Hongze/14/05 H11N9 A/duck/Hong Kong/MPS180/03 H4N6 (both viruses 97.2)

^a^Nucleotide sequence identity within each gene segment of our isolates; ^b^identity with genes of viruses from GenBank.

**Table 6:** Comparison of avian H1N1 strain with pandemic and seasonal flu strains.

Virus	Aomori/422/07 H1N1 identity (%)		
	Nucleotide	Amino acid	Nucleotide
Segment 4 (HA)			
A/Brevig Mission/1/18 H1N1	67.8	92.1	67.1
A/Mexico city/001/09 H1N1	75.3	82.5	74.7
A/Japan/AF07/08 H1N1	76.4	83.6	76.4

Segment 6 (NA)			
A/Brevig Mission/1/18 H1N1	84.5	92.3	84.5
A/Mexico city/001/09 H1N1	86.5	90.0	86.5
A/Japan/AF07/08 H1N1	79.3	84.9	79.5
